# The Relationship Between Meal Composition and the Body Composition of Wroclaw Medical University Students

**DOI:** 10.3390/nu18101602

**Published:** 2026-05-18

**Authors:** Aleksandra Jaremków, Iwona Markiewicz-Górka, Krzysztof Kraik, Krystyna Pawlas, Rafał Poręba, Małgorzata Poręba, Paweł Gać

**Affiliations:** 1 Department of Environmental Health, Occupational Medicine and Epidemiology, Wroclaw Medical University, Mikulicza-Radeckiego 7, 50-345 Wroclaw, Poland; iwona.markiewicz-gorka@umw.edu.pl (I.M.-G.); krzysztof.kraik@student.umw.edu.pl (K.K.); krystyna.pawlas@umw.edu.pl (K.P.); pawel.gac@umw.edu.pl (P.G.); 2 Department of Biological Principles of Physical Activity, Wroclaw University of Health and Sport Sciences, al. Ignacego Jana Paderewskiego 35, 51-612 Wroclaw, Poland; rafal.poreba@awf.wroc.pl (R.P.); malgorzata.poreba@awf.wroc.pl (M.P.)

**Keywords:** BMI, body composition, diet, young adults

## Abstract

Background/Objectives: Appropriate nutrition is a foundation for maintaining good health. Especially for young people, it determines the proper growth and development. The aim of the study was to investigate the relationship between the dietary makeup of young adults and their body composition parameters. Methods: A total of 297 students of Wroclaw Medical University participated in the study. A questionnaire was administered to assess meal composition, and body composition, phase angle, and basal metabolic rate (BMR) were evaluated. Results: The greatest differences were observed in the consumption of grains, animal protein, and dairy products. Meals consumed by males contained mostly animal protein and grain products, whereas females’ meals contained more fruit and vegetables. Correlations were observed between dinner, supper and the extracellular water/intracellular water (ECW/ICW) ratio, fat, water, and muscle body content, with r~ ±(0.134–0.163), and between the second breakfast and body mass index (BMI), and visceral fat level (respectively: −0.118; −0.149). Multivariate analysis indicated that consuming a balanced dinner has a significant impact on maintaining the appropriate proportions of body composition. Conclusions: Analysis of the results suggests that proper composition of meals is associated with a lower BMI, reduced fat content, increased muscle mass, and better body hydration.

## 1. Introduction

According to the Polish National Center for Nutrition Education and WHO recommendations, proper nutrition has a significant impact on maintaining good health. Appropriate nutrition is one of the most important factors in the growth and development of the human body at a young age. Furthermore, at older age, the appropriate diet reduces morbidity and mortality from several chronic diseases, including cardiovascular diseases, neoplastic diseases, and diabetes mellitus (leading causes of death worldwide). Moreover, the proper diet contributes to life extension [1,2]. Considering these beneficial effects, the Pyramid of Healthy Nutrition and Physical Activity was developed. According to its principles, vegetables and fruit should be the type of food consumed in the largest amount, while fats, especially animal fats, containing saturated fatty acids, should be the most limited. 4–5 meals should be consumed daily, regularly at fixed times every day, and there should be 3–4 h-long intervals between them. Among grain products, whole-grain options should be chosen. Two glasses of milk, kefir, or yogurt, and, less frequently and in smaller quantities than the above, rennet cheeses, should be consumed daily. Meat intake should be reduced in favor of fish, legumes, and eggs. Instead of sweets, fruits and nuts should be eaten, and herbs should be used instead of salt. At least 1.5 L of water should be drunk daily, and alcohol should be completely avoided [2].

Besides the guidelines for healthy eating, attention should also be paid to the Healthy Eating Plate [3], developed by Harvard Medical School. According to experts, a well-balanced meal should consist of: half a plate of vegetables and fruit, one quarter of whole-grain products and one quarter of a healthy protein source. Apart from these products, a proper source of water and healthy fats should be provided. In turn, the latest guidelines developed by the U.S. Department of Health and Human Services and the U.S. Department of Agriculture emphasize the role of protein and full-fat dairy products. They indicate that in nutrition, the priority is primarily the quality of the products consumed, not their proportions (the least processed food possible) [4].

Numerous methods and indicators exist to assess diet quality. Dietary assessment often involves the analysis of meal plans using either theoretical or laboratory methods. Theoretical methods are divided into qualitative, which characterize the meals, and quantitative, which calculate the nutritional value and amounts of products relative to dietary standards. Laboratory methods involve the determination of protein, fat, fiber, and ash content and the calculation of carbohydrate content. Results from theoretical and laboratory methods are then compared. Among the most commonly used methods are qualitative point-based dietary assessments (e.g., developed by Bielińska or Starzyńska [5]). Some researchers evaluate meals based on the presence of specific nutrients (e.g., Catalani et al. [6]). In the National Survey of Dietary Habits and Nutritional Status of the Polish Population [7], conducted by the National Institute of Public Health PZH—National Research Institute in 2019–2020, two types of data were collected: data on the products consumed and their quantity, and data on food composition (energy value and nutrient content), and then the food was classified accordingly. The methodology in the cited study followed European Food Safety Authority (EFSA) guidelines. Other researchers have used methods developed by themselves, including author-designed questionnaires about dietary habits [8,9,10,11] or used previously available questionnaires, such as the Food Frequency Questionnaire (FFQ) or the Dietary Habits and Nutrition Beliefs Questionnaire (KomPAN) [12,13,14]. No single method can be considered the most reliable. Therefore, multiple methods are used, and new ones are being developed.

The Polish dietary model recognizes three main meals: breakfast, dinner, and supper. Second breakfast and afternoon snack are considered complementary meals. Breakfast is consumed in the morning (6:00–9:00 a.m.), dinner around 12:00–3:00 p.m. (4:00 p.m.), and supper in the evening (6:00–9:00 p.m.). According to the most recent report on the Polish population, the majority of adults do not eat regularly, limiting themselves to three meals per day, but they snack between meals. Their eating patterns significantly deviate from recommendations: vegetables, fruit, dairy products, and fish are consumed too infrequently, while the intake of red meat, sweets, soft drinks, and butter is excessive [7]. Studies conducted among young people in Poland indicate that diet quality declines with age, with dietary errors and poor eating habits becoming more frequent [15,16]. A long-term American longitudinal study of adolescents—young adults’ nutrition revealed the improvement in the consumption of vegetables and whole-grain products with age, at the expense of fruit and dairy products [17].

Up to this time, only a relatively small number of studies investigating the association between diet quality and body composition have been performed. Among the numerous parameters, body mass index (BMI), body weight, body fat percentage, and waist circumference are the ones analyzed the most commonly. The increase in these parameters typically correlates with poor dietary behaviors and low-quality diets [18,19,20]. However, comprehensive analyses of the relationship between body composition and dietary patterns are still lacking.

The current study was performed because of the discrepancies concerning diet quality, the diversity of used research methods, and the limited data linking these issues with body composition parameters in the currently available studies. It aims to assess the quality of meals consumed by young people in terms of their balance and to determine whether there is an association between meal composition and body composition parameters in the population of students.

## 2. Materials and Methods

### 2.1. Study Design

The sample size was calculated using the minimum sample size formula. Selection criteria: population size (number of students in medical faculties of a large academic center (Wroclaw Medical University)—approximately 6000), maximum error: 6%, confidence level: 95%. The required minimum sample size was 256.

All students (from the Medical University of Wroclaw) with no contraindications to measurement with a body composition analyzer were invited to the study. Approximately 300 individuals expressed interest in participating, of whom 297 students of Wroclaw Medical University were qualified (the Faculty of Medicine and the Faculty of Medicine and Dentistry). Students who returned incomplete questionnaires and had measurement contraindications were not included. It consisted of two parts: a questionnaire and a measurement of body composition, basal metabolic rate (BMR), and phase angle (PA).

This study was approved by the Bioethics Committee at the Wroclaw Medical University in Poland (KB-524/2017; KB-46/2018).

### 2.2. Questionnaire Survey

Students were asked to complete an author’s questionnaire, which included basic sociodemographic questions, including age, sex, and place of residence, and questions about the composition of the meals they consume daily: “What meals you eat every day? What dishes do they most often consist of?”; “What do you drink most often during the day?” Exact questions regarding the composition of meals and beverages consumed daily is presented in Supplementary Materials. (Meals-Polish dietary model: main meals—breakfast, dinner, supper and complementary meals—second breakfast and afternoon snack) Table 1.

### 2.3. Assessment of Meal Nutritional Adequacy

The meal scoring (Table 1) was based on the presence of basic food groups, in accordance with healthy eating guidelines: Healthy Eating Plate [3], including dairy products as a separate category (in reference to the American guidelines emphasizing the role of dairy products [4]). The analysis covered the following meals: first and second breakfast, dinner, afternoon snack, and supper. For each meal (first breakfast, second breakfast, dinner, afternoon snack, supper), the presence of individual food groups was scored, 1 point for each group, including grains, animal protein, dairy products, and vegetables and fruit, while the presence of fast food or sweets reduced the score by 1 point each. A maximum of 4 points could be scored for each meal. A properly composed meal was defined as one scoring ≥3 points. For the assessment of overall daily dietary quality, at least 3 healthy meals per day were considered satisfactory (3 meals ≥ 3 points each). Additionally, beverage consumption was scored as follows: 1 point was scored for coffee, tea, natural fruit juice, and water, and 1 point was removed for energy drinks and cola drinks. We considered a score of ≥1 point (including water– mandatory, as water is the healthiest drink [21,22]) to be satisfactory.

### 2.4. Body Composition Analysis

The participants’ body composition was measured, and their BMR and phase angle were calculated using a TANITA MC-780MA device. The measurements were performed using the bioelectrical impedance analysis (BIA) method.

According to the device’s instructions, the participants attended the examination wearing possibly light clothing, barefoot, without jewelry, and with empty pockets. Additionally, the participants must have met the following criteria regarding the preparation for measurement:

- at least 3 h after getting out of bed,
- at least 3 h after the last meal,
- at least 12 h after the last intense physical exercise,
- at least 12 h after last alcohol consumption,
- after emptying the bladder,
- and for women—excluding the time of menstruation and pregnancy.

Participants with pacemakers or other implantable devices and medical implants were excluded from the study due to their significant effect on the body composition monitor, which increases the risk of measurement error.

### 2.5. Statistical Analysis

The statistical analysis was performed using “Statistica 13.0 for Windows” software. To demonstrate differences in composition between meals, the nonparametric Cochran’s Q test was used. The composition of individual meals and their quality were also compared by sex using the Chi-square test. Anthropometric and body composition parameters were analyzed using Student’s t-test, and the Mann–Whitney U test when variables did not follow a normal distribution, assessing differences by sex and by quality of nutrition. Spearman correlations were performed to examine associations between the number of basic food groups in individual meals and anthropometric and body composition parameters. A multivariable analysis of the impact of the quality of main, well-balanced meals, including first breakfast, second breakfast, dinner, afternoon snack, and supper, on anthropometric and body composition parameters was conducted using multiple regression with a stepwise method. To identify which factors characterizing the study population influenced diet quality, logistic regression analysis was performed. A significance level of *p* < 0.05 was adopted for all statistical calculations.

## 3. Results

### 3.1. Characteristics of the Studied Population

Among the study participants (297 students), the majority were approximately 21 years old and in their second year of study, while the remainder were in their third and fourth years. A comparable number of females and males were examined, with a slight majority of females (58.2%). Almost all participants lived in Wrocław and studied at the Faculty of Medicine. Only 13 students studied at the Faculty of Dentistry. Most students originated from large cities, while others came to study from smaller cities, towns, and villages in similar proportions. About two-thirds of participants rented a room or an apartment. Fewer students lived in a dormitory or family houses. The surroundings of the current place of residence of more than half of the participants were reported as peaceful (Table 2).

### 3.2. Diversification of the Composition of Individual Meals

The composition of individual meals differed significantly (Table 3). The greatest differences were observed in the consumption of grains, animal protein, and dairy products. A similar percentage of students consuming these products was observed only between the first breakfast and dinner (grains), the first breakfast and supper (animal protein), and dinner and the afternoon snack (dairy products). Participants most often ate vegetables and fruits for dinner and a second breakfast. For the remaining meals, no significant differences in their consumption were observed. Fast food and sweets were consumed the least frequently. A slightly higher intake of fast food was observed at dinner when compared with other meals. None of the participants reported eating sweets for dinner, while they were most commonly consumed during the afternoon snack. A low intake of sweets was also noted during other meals.

### 3.3. Meal Composition and Sex

**Figure 1:**
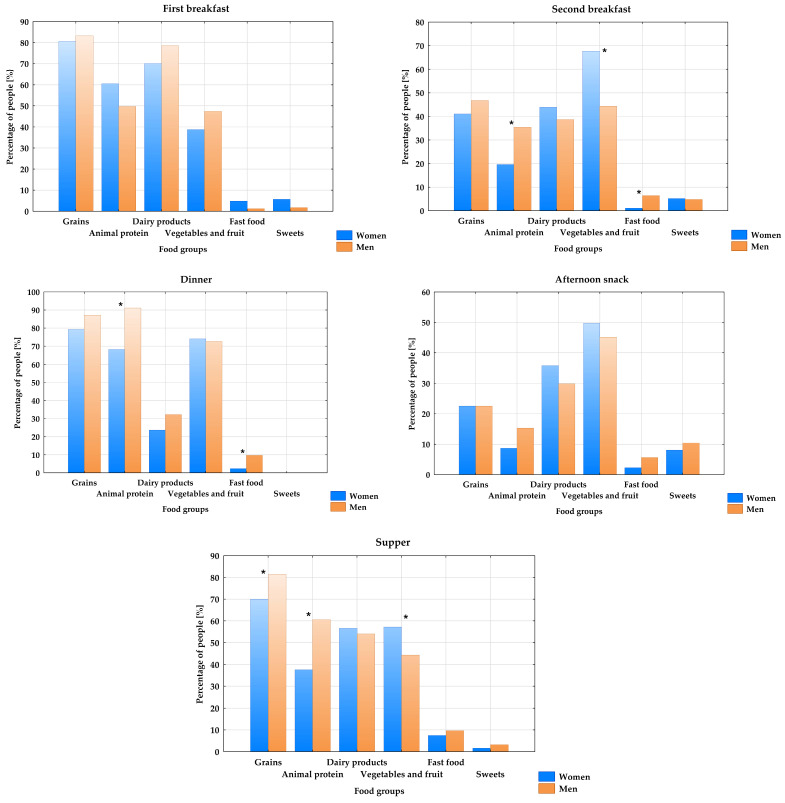
Percentage of students consuming specific food groups by sex. * *p* < 0.05 men vs. women.

The composition of the meals differed significantly between males and females, with the exception of the first breakfast and the afternoon snack (Figure 1). Meals consumed by males significantly more often contained animal protein (second breakfast, dinner, supper), grains (supper), and fast food than the meals consumed by females. Furthermore, females’ meals were richer in vegetables and fruits than males’ meals (second breakfast, supper).

### 3.4. Analysis of the Diet Quality

**Figure 2:**
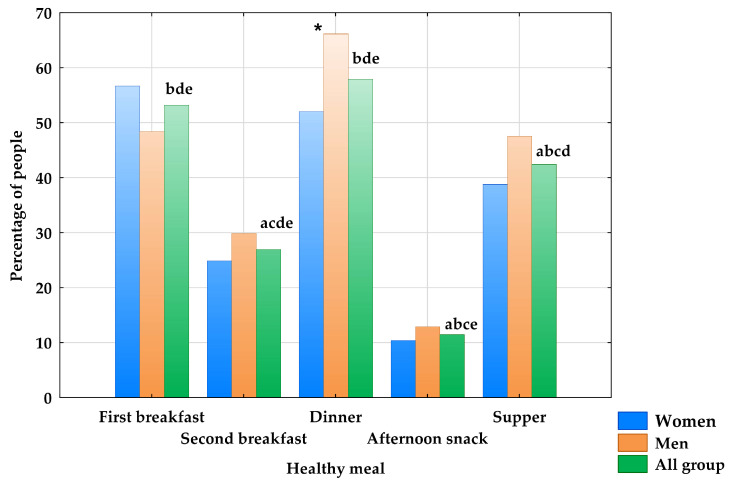
Percentage of students consuming well-balanced meals (≥3 points) by sex. * significant differences between men and women; significant difference with: ^a^ first breakfast; ^b^ second breakfast; ^c^ dinner; ^d^ afternoon snack; ^e^ supper.

In the analysis of differences in meal quality (Figure 2; well-balanced meals are defined as meals with ≥3 points), significant differences were observed between all meals except for the first breakfast and dinner. Dinner was the meal that was most often well-balanced, while the afternoon snack, which was very often skipped by the participants, was most rarely well-balanced. Apart from dinner (men—66.1% vs. women—52.0%, *p* < 0.05), no significant sex-related differences in diet quality were found.

Taking into account the quality of students’ daily nutrition, only one third of participants (31.7%) ate adequately (at least 3 properly composed meals during the day). As many as three-quarters of the participants (76.1%) quenched their thirst with appropriate beverages.

### 3.5. Differences in Body Composition and Anthropometric Parameters

Anthropometric and body composition parameters in the study group differed significantly by sex (Table 4). Males had significantly higher height, body weight, total body water, total muscle mass, BMI, PA (phase angle), BMR, fat-free mass (FFM), bone mass and visceral fat level, while the females had a significantly higher total body fat content and extracellular water/intracellular water (ECW/ICW) ratio. No significant differences were observed in anthropometric parameters or body composition parameters between individuals who ate at least three well-balanced meals per day and the other participants (*p* > 0.05).

### 3.6. Analysis of the Relationship Between the Number of Ingredients in Individual Meals and Students’ Anthropometric and Body Composition Parameters

Statistically significant positive correlations were observed between the number of basic food groups consumed at dinner and supper and the percentage of body water and muscle, and negative correlations were found between the number of basic food groups consumed at dinner and supper and body fat percentage and the ECW/ICW ratio. Moreover, the number of ingredients in the second breakfast negatively correlated with BMI and visceral fat level (Table 5).

### 3.7. Multivariate Analysis of the Effect of Main Meals on Anthropometric and Body Composition Parameters

In the presented model (Table 6), the main meals were defined as: (1) breakfasts (first and second), (2) dinner, and (3) afternoon snack + supper. Based on multivariate analysis, it was found that consuming a well-balanced dinner was significantly associated with an increase in body water and muscle content and a decrease in fat percentage and the ECW/ICW ratio. Moreover, a negative correlation was observed between breakfast and the PA value.

Based on multivariate analysis, it was found that consuming a well-balanced dinner was significantly associated with an increase in body water and muscle content and a decrease in fat percentage and the ECW/ICW ratio.

### 3.8. Influence of Various Characteristics of the Studied Population on the Quality of Nutrition

Among the various factors characterizing the study population, year of study, type of housing, and its surroundings had a significant impact on diet quality (defined as at least three well-balanced meals during the day). Third- and fourth-year students ate better-balanced meals than the students of the lower years. Having one’s own or rented room or apartment and living in a quiet neighborhood also had a beneficial effect on diet quality. No other significant associations were observed in this regard in the case of other factors (*p* < 0.05). The full results are presented in Table 7.

## 4. Discussion

The results of our study indicate substantial variability in the composition of meals consumed by students over the course of the day. Primarily, this variability concerned the presence of grains, animal protein, and dairy products, which predominated at dinner/ first breakfast. It was found that grain products predominated in most meals. This is consistent with Asian study findings [23], stating that consumption of such a food group predominated throughout the day (five times a day), while fruits were consumed only once or twice a week. In our study, almost half of the students reported consuming both fruits and vegetables with every meal; notably, this proportion rose to three-quarters of participants at dinner. Products containing animal protein were also most frequently consumed during dinner. This is the main meal of the day, so it should be as satiating as possible (presence of animal protein) [24]. An Australian study also showed that products containing animal protein, especially meat, are mostly consumed at dinner [25]. However, in Australia, the dinner is consumed at late afternoon or evening, which is at later hours than in Poland, and therefore it functions as a combined dinner and supper. In many English-speaking countries, the typical meal pattern is breakfast consumed in the morning, lunch consumed about 12:00–14:00, which is a light meal such as a salad or sandwich, and dinner, consumed about 18:00, being the main meal of the day. Additionally, there may be an “afternoon tea”, which is a small afternoon snack, and “supper”, a lighter evening meal [26]. In our study, we tried to match the meaning of individual Polish meal types to their English equivalents in terms of their role in the daily eating pattern.

The students surveyed in this study consumed dairy products mostly at breakfast. This is a common preference. Students’ breakfasts in various countries have dairy products as the primary component [27,28], possibly because they are widely available and require the least amount of time for meal preparation [28]. It should be emphasized that only a small percentage of students reported consuming fast food and sweets (8–9%). However, numerous studies [29,30] indicate persistent harmful dietary patterns among young people and an excessive frequency of unhealthy food consumption. Therefore, it can be assumed that, because the participants of the current study were medical students, they possessed greater knowledge and awareness of healthy behaviors.

Certain differences in the composition of individual meals were observed depending on the sex of the participants. Males more frequently than females consumed dishes containing animal protein and grains, and were also more likely to consume fast food, while females’ diets were richer in vegetables and fruit. Women generally tend to have a stronger belief in the benefits of healthy eating, e.g., inclusion of vegetables and fruit in meals, and in maintaining an appropriate body shape than men do [31]. Numerous studies [32,33] also confirmed the existence of a strong so-called “meat-masculinity” association, due to which men consume more animal protein than women. However, the findings of the current study regarding the presence of cereal products in meals differ from other studies [34,35], which report that women more often consume grain products, especially whole-grain ones. The discrepancy may result from the fact that grain products were not categorized in our study.

In terms of nutritional completeness, the dinner and the first breakfast were the most well-balanced meals of the day. Generally, meals such as first breakfast, dinner, and supper were of higher nutritional quality than the second breakfast and the afternoon snack. This is understandable because the three main meals listed above form the basis of daily nutrition, while the other two have a supplementary role. Significant sex differences were observed only for dinner, as it was more frequently well-balanced among males than among females. This is contrary to the common belief that women pay greater attention to appropriate eating patterns [31,36]. However, considering that dinner is the main meal of the day and that men typically have higher caloric needs, resulting from greater body weight and muscle mass, dinner for men may have been better balanced. Moreover, men more often skip or neglect breakfast when compared to women and therefore may place greater attention on the quality of the dinner they consume [37].

Regarding overall daily diet quality, only one-third of respondents consumed at least three well-balanced meals during the day, while three-quarters of respondents chose appropriate beverages to quench thirst. A study of a student population of the Medical University of Białystok similarly found that students primarily selected appropriate thirst-quenching beverages, mostly water, followed by tea and coffee, and only 5% consumed soft drinks [38]. Both coffee and tea, due to the content of health-promoting components, such as the aflavins, catechins, and polyphenols, are reported to have preventive and therapeutic effects for a range of conditions, including obesity, metabolic syndrome, type 2 diabetes mellitus, and cardiovascular diseases [39]. Consequently, they are considered healthy beverages, albeit within certain limitations of daily consumption, being 3–4 cups for coffee, and 4–5 cups for tea [40,41]. Although young people tend to drink appropriate beverages, overall dietary quality throughout the day remains suboptimal. In a 2021–2022 report on young Poles, similarly to the current study, it was found that only 31% of them regularly consumed balanced meals [42].

The anthropometric and body composition parameters presented in Table 4 varied by sex, which is expected given the anatomical and physiological differences between men and women.

Analysis of the association between the number of components, representing basic food groups, in individual meals and anthropometric and body composition parameters showed that the more nutritionally complete the consumed meal, the more favorable the effects on the body. In the case of a second breakfast, there is a decrease in visceral fat and BMI. The better the dinner and supper composition, the lower the fat content and the ECW/ICW ratio, and the higher the body water and muscle content were recorded. Moreover, the multivariate analysis confirmed these associations for dinner. Currently available literature lacks studies demonstrating these types of relationships, and thus, findings of this study may provide valuable data regarding the importance of individual meals for health. To date, only breakfasts, being the first meal of the day, have been analyzed with respect to such outcomes. However, these studies mostly included the child population only. The breakfast consumption has been shown to prevent the development of obesity [43], higher-quality breakfasts are associated with lower BMI [44], and regular breakfast consumption correlates with low body fat content [45]. We found that over half of the students ate a healthy breakfast. For comparison, in the study by Kawalec et al. [46], in two-thirds of the examined Polish school-age children, this meal was balanced. In this case, a point-based meal assessment method was also used, but according to the criteria developed by Catalani et al. [6], which took into account the presence of nutrients such as water, carbohydrates, protein, dairy products, fiber, vitamins, minerals, and added simple sugars. The differences may also result from the fact that parents take care of children’s proper nutrition, while in the case of young adults, this responsibility usually falls on them.

Studies on adult populations have also shown negative correlations between good breakfast quality and abdominal obesity, measured with waist-hip ratio (WHR) [47] and BMI [48]. According to research conducted by Saintila et al., medical students are more likely than non-medical students to eat regular and better-quality breakfasts and have a lower BMI [49]. However, in the case of other meals, no studies were performed. The correlations observed in the current study indicate the significant roles of the following meals: second breakfast, dinner, and supper. Interestingly, the observed associations relate to the second breakfast rather than the first breakfast, even though the first breakfast was better balanced. Second breakfasts were richer in vegetables and fruit, which are sources of fiber, essential vitamins, and minerals, indicating the importance of these components for health, maintenance of proper metabolism, and consequently, proper body silhouette [50]. This may be confirmed by the results of Chinese studies analyzing the dietary patterns in students of medical faculties and their body composition, which obtained a correlation between fruit consumption and fat tissue content. This means that fruit consumption may be indicative of an overall better dietary profile [51].

The percentages of fat, water, and muscle body content are parameters closely related to each other. The ECW/ICW ratio is an indicator of correct cellular function and hydration and is also a factor considered in the assessment of muscle strength [52]. In turn, the level of visceral fat is important for assessing health risk. It accumulates in the abdominal cavity (not under the skin), surrounding, among other places, the liver and pancreas. It is metabolically active and produces pro-inflammatory substances; therefore, its excessively high levels are dangerous to health [53]. The associations observed in the current study, especially between dinner and supper and the parameters described before, may indicate the importance of these meals for maintaining appropriate proportions of basic body constituents such as water, adipose and muscle tissue. The Spanish research shows that the deterioration in the quality of nursing students’ diet had a negative impact on their body composition, especially fat and muscle tissue [54]. The multivariate analysis we performed also revealed the negative correlation between PA and the composition of first and second breakfasts, which consisted mainly of grain and dairy products for the first breakfast, and fruits and vegetables for the second breakfast. The available literature [53,55] reports positive associations between PA and consumption of products with high protein content, mainly meat. Therefore, it is plausible that the negative correlation observed in this study (PA vs breakfast composition) may be caused by lower intake of such high-protein foods at both breakfasts. PA is a parameter regarding the cellular nourishment and proper functioning, including the maintenance of cell membrane integrity, efficiency of energetic processes, and proteolysis. Consumption of meat products supplies the body mainly with energy and structural nutrients, which explains their beneficial effect on PA values [55].

The current study also found that older students, attending third- and fourth-year of the faculty, who rented or owned a room or an apartment in a quiet neighborhood, had a better-balanced diet than younger students living in dormitories, family homes or noisy surroundings. The influence of year of study on diet quality can be explained by increasing knowledge and awareness over time, particularly among students in medical fields. An analysis conducted by Belgian researchers revealed that dormitory residence often led to negative dietary behaviors among students. However, contrary to findings of the current study, it was found that living in the family home beneficially influenced the diet quality, including more frequent consumption of vegetables and fruit and greater parental control over eating [56]. Other studies also confirm that eating in noisy environments more often leads to unfavorable dietary behaviors, such as greater consumption of unhealthy products, and less conscious food choices [57,58].

The strength of the study is the large sample size, with a comparable number of women and men, which increases the reliability and universality of the results. This is the first study to analyze in such detail the relationship between the composition/quality of consumed meals and body composition, which provides a foundation for further research focused on the importance of nutrition (specific meals/main products) for maintaining the correct body parameters/proportions (water/fat/muscle).

A limitation of our study is that it is based on participants’ self-reported diets (the composition of daily meals). The information provided by respondents is difficult to verify. In addition, the assessment of the meals consumed was only qualitative. Therefore, in future studies, it would be worthwhile to use more precise nutrition assessment methods (e.g., quantitative) to make the results more reliable. The study was conducted in a group of students in a narrow age range, which makes it difficult to extrapolate the obtained results to other groups/the entire population. Another limitation may be the research method used—bioelectrical impedance, which is an estimation method (based on algorithms) rather than a direct measurement. Furthermore, it is very sensitive to body hydration levels (margin of error—2%). Therefore, in our study, participants were instructed on how to prepare for the test to ensure accurate results. A limitation of the study is also the lack of consideration of other lifestyle factors in the analysis, which could have provided a more comprehensive picture of the relationships between body composition parameters and other variables in this area. It should be emphasized that lifestyle includes many different factors, the analysis of which would be very extensive. Nevertheless, this constitutes a premise for undertaking further research.

## 5. Conclusions

The analysis of the dietary habits of students at the Wroclaw Medical University showed that not every student followed the principles of a healthy lifestyle. Only one-third of the participants paid attention to consuming properly composed meals throughout the day. However, as many as three-quarters of the participants quenched their thirst with appropriate beverages. Therefore, it cannot be unequivocally stated that the overall quality of their diet was inadequate, particularly since they rarely consumed sweets and fast food. Of all the meals analyzed, the first breakfast and dinner stood out for their good composition, and these are the main meals forming the basis of daily nutrition. There were dimorphic differences in the composition of the meals. Among women, consumption of vegetables and fruits prevailed, while men, due to their higher caloric needs, consumed animal protein and grain products.

The analysis of associations between the composition of individual meals consumed during the day and body composition parameters confirmed the health significance of meals such as breakfast, dinner, and supper. A balanced breakfast (especially the second breakfast) could be associated with a lower BMI and a decrease in visceral fat, which was crucial for maintaining a healthy figure. In turn, consuming a well-composed dinner and supper is correlated with reduced fat content, increased muscle tissue, and improved hydration. This is the first study in which these associations were observed.

The results of our study may present a guide for young people towards more consciously composed meals to obtain specific health benefits.

## Figures and Tables

**Table 1:** Meal scoring.

Type of Meal	Grains	Animal Protein	Dairy Products	Vegetables and Fruit	Fast Food	Sweets
First breakfast						
Cereal flakes with milk	1		1			
Sandwiches	1	Depending on the sandwiches’ composition (1)	Depending on the sandwiches’ composition (1)	Depending on the sandwiches’ composition (1)		
Vegetables/fruit (e.g., salad)				1		
Homogenized cheese/yogurt/kefir			1			
Egg		1				
Fast food, e.g., hamburger/hot dog					−1	
Others, e.g., sweets						−1
Second breakfast						
Sandwiches	1	Depending on the sandwiches’ composition (1)	Depending on the sandwiches’ composition (1)	Depending on the sandwiches’ composition (1)		
Vegetables/fruit (e.g., salad)				1		
Homogenized cheese/yogurt/kefir			1			
Egg		1				
Fast food, e.g., hamburger/hot dog					−1	
Others, e.g., sweets						−1
Dinner						
Soup				1		
Stuffed dumplings	1		1			
Meat dishes		1				
Fish		1				
Potatoes				1		
Rice	1					
Pasta	1					
Fruit/vegetable salad				1		
Fast food, e.g., gyros/pita/hamburger/hot dog					−1	
Others, e.g., sweets						−1
Afternoon snack						
Sandwiches	1	Depending on the sandwiches’ composition (1)	Depending on the sandwiches’ composition (1)	Depending on the sandwiches’ composition (1)		
Vegetables/fruit (e.g., salad)				1		
Homogenized cheese/yogurt/kefir			1			
Fast food, e.g., hamburger/hot dog					−1	
Others, e.g., sweets					−1	
Supper						
Sandwiches	1	Depending on the sandwiches’ composition (1)	Depending on the sandwiches’ composition (1)	Depending on the sandwiches’ composition (1)		
Vegetables/fruit (e.g., salad)				1		
Pasta with toppings	1	Depending on the toppings (1)	Depending on the toppings (1)	Depending on the toppings (1)		
Rice with toppings	1	Depending on the toppings (1)	Depending on the toppings (1)	Depending on the toppings (1)		
Homogenized cheese/yogurt/kefir			1			
Fast food, e.g., hamburger/hot dog					−1	
Others, e.g., sweets						−1

(1): 1 point is awarded depending on the sandwiches’ composition.

**Table 2:** Characteristics of the studied population (*n* = 297).

Characteristics	Mean ± SD or *n* (%)
Age (years)	20.9 ± 1.4
Sex (females)	173 (58.2)
Year of study:	
II	237 (79.8)
III	59 (19.9)
IV	1 (0.3)
Faculty:	
Medicine	284 (95.6)
Dentistry	13 (4.4)
Place of residence—current:	
Wrocław	289 (97.3)
outside Wrocław	8 (2.7)
Place of residence—origin:	
Village	65 (21.9)
Small Town ^a^	67 (22.5)
City ^b^	57 (19.2)
Big city ^c^	108 (36.4)
Type of apartment:	
Dormitory	49 (16.5)
Rented room	91 (30.6)
Rented/owned apartment	100 (33.7)
Family house	57 (19.2)
The surroundings of the current place of residence:	
Heavy traffic; noisy	117 (39.4)
Proximity to recreational areas; peaceful	162 (54.5)
Other	18 (6.1)

^a^ with fewer than 50,000 inhabitants; ^b^ with 50,000–100,000 inhabitants; ^c^ with over 100,000 inhabitants.

**Table 3:** Diversity of meal composition in the study population.

Meal	Students Who Consumed the Following Food Groups [*n* (%)]					
	Grains ^a^	Animal Protein ^b^	Dairy Products ^c^	Vegetables and Fruit ^d^	Fast Food ^e^	Sweets ^f^
First breakfast	244 (82.2)	161 (54.2)	223 (75.1)	130 (43.8)	8 (2.7)	10 (3.4)
Second breakfast	129 (43.4)	78 (26.3)	124 (41.8)	172 (57.9)	10 (3.4)	15 (5.1)
Dinner	245 (82.5)	231 (77.8)	81 (27.3)	218 (73.4)	16 (5.4)	0 (0.0)
Afternoon snack	67 (22.6)	34 (11.5)	99 (33.3)	142 (47.8)	11 (3.7)	27 (9.1)
Supper	222 (74.8)	140 (47.1)	165 (55.6)	154 (51.9)	25 (8.4)	7 (2.4)

Significant differences (*p* < 0.05) between all meals except: ^a^ first breakfast vs. dinner; ^b^ first breakfast vs. supper; ^c^ dinner vs. afternoon snack; ^d^ first breakfast vs. supper, second breakfast vs. supper, afternoon snack vs. supper; ^e^ first breakfast vs. second breakfast, first breakfast vs. dinner, first breakfast vs. afternoon snack, second breakfast vs. dinner, second breakfast vs. afternoon snack, dinner vs. afternoon snack, dinner vs. supper; ^f^ first breakfast vs. second breakfast, first breakfast vs. supper, second breakfast vs. afternoon snack, second breakfast vs. supper.

**Table 4:** Body composition and anthropometric parameters of the study group divided by sex.

Parameter	Total [Mean ± SD]	Men [Mean ± SD]	Women [Mean ± SD]	Test Value ^a^
Height (cm)	172.2 ± 9.0	180.1 ± 6.6	166.5 ± 5.6	19.0 ^b^
Body weight (kg)	65.1 ± 12.3	75.0 ± 9.8	58.0 ± 8.4	16.3 ^b^
Fat percentage (%)	19.9 ± 6.2	15.7 ± 4.7	23.0 ± 5.4	−12.3 ^b^
Water percentage (%)	57.9 ± 4.7	61.0 ± 4.0	55.6 ± 3.9	11.8 ^b^
Muscle percentage (%)	76.0 ± 5.9	80.1 ± 4.4	73.1 ± 5.1	12.4 ^b^
BMI (kg/m^2^)	21.8 ± 2.9	23.1 ± 2.4	20.9 ± 2.9	6.8 ^b^
ECW/ICW	0.678 ± 0.042	0.652 ± 0.039	0.696 ± 0.033	−10.5 ^b^
PA (°)	6.0 ± 0.7	6.6 ± 0.5	5.5 ± 0.5	18.2 ^b^
BMR (kJ)	6627.5 ± 1237.6	7857.4 ± 856.3	5746.0 ± 489.2	14.2 ^c^
FFM (kg)	52.1 ± 10.7	63.0 ± 6.8	44.3 ± 4.0	14.4 ^c^
Bone mass (kg)	2.6 ± 0.5	3.1 ± 0.3	2.3 ± 0.2	14.3 ^c^
Visceral fat level [1–59]	1.7 ± 1.4	2.4 ± 1.6	1.3 ± 0.9	7.1 ^c^

BMI—body mass index; ECW/ICW—extracellular water/intracellular water ratio; PA—phase angle; BMR—basal metabolic rate; FFM—fat-free mass; ^a^ at the level of statistical significance *p* < 0.002; ^b^ Student’s *t*-test; ^c^ Mann–Whitney U test.

**Table 5:** Statistically significant correlations between the number of basic food-group components in individual meals and students’ anthropometric and body composition parameters.

Spearman’s Correlation (r)					
Anthropometric and Body Composition Parameters	First Breakfast	Second Breakfast	Dinner	Afternoon Snack	Supper
Fat percentage	-	-	−0.163	-	−0.132
Water percentage	-	-	0.158	-	0.121
Muscle percentage	-	-	0.163	-	0.134
BMI	-	−0.118	-	-	-
Visceral fat level	-	−0.149	-	-	-
ECW/ICW	-	-	−0.153	-	−0.137

BMI—body mass index; ECW/ICW—extracellular water/intracellular water ratio.

**Table 6:** Effect of main meals on anthropometric and body composition parameters.

Meals	Fat Percentage ^a^		Water Percentage ^b^		Muscle Percentage ^c^		ECW/ICW ^d^		PA ^e^	
	*β*	*p* Value	*β*	*p* Value	*β*	*p* Value	*β*	*p* Value	*β*	*p* Value
Breakfasts (first and second)	0.085	0.156	−0.076	0.206	−0.085	0.155	0.059	0.311	−0.161 *	0.008
Dinner	−0.138 *	0.021	0.133 *	0.026	0.138 *	0.020	−0.119 *	0.042	0.087	0.145
Afternoon snack and supper	−0.066	0.280	0.067	0.273	0.067	0.275	-	-	0.039	0.517

^a,b,c,d,e^ separate regression analyses; ECW/ICW—extracellular water/intracellular water ratio; PA—phase angle; * statistically significant.

**Table 7:** Odds ratios (OR) and 95% confidence intervals (CIs) for the good quality of diet relative to various characteristics of the studied population.

Factors	OR	95% CI	*p*
Sex	0.79	0.47–1.34	0.383
Year of study	3.49	1.91–6.39	0.000 *
Faculty	0.56	0.12–2.68	0.462
Family place of residence (origin)	1.00	0.79–1.28	0.982
Current place of residence	1.66	0.31–8.91	0.552
Type of apartment	1.35	1.01–1.82	0.045 *
Surroundings of the current place of residence	0.72	0.55–0.96	0.024 *

* statistical significance.

## Data Availability

The data presented in this study are available on request from the corresponding author upon reasonable request due to ethical restrictions.

## Supplementary Materials

The following supporting information can be downloaded at: https://www.mdpi.com/article/10.3390/nu18101602/s1, Exact questions regarding the composition of meals and beverages consumed daily.
