# Supplementary material for: The Relationship Between Meal Composition and the Body Composition of Wroclaw Medical University Students
Source: Nutrients. 2026 May 18;18(10):1602. doi: 10.3390/nu18101602 (PMC13209570; doi:10.3390/nu18101602)
Supplement: Supplementary file 1 [file nutrients-18-01602-s001.zip › nutrients-4288806-supplementary.pdf]

What do you drink most often during the day?

a) coffee

b) tea

c) cola drinks

d) energy drinks

e) natural fruit juice

f) water

g) others………………………………

What meals you eat every day? What dishes do they most often consist of?:

a) First breakfast:

a.1) cereal flakes with milk

a.2) sandwiches with ……………………

a.3) vegetables/fruit (e.g., in a salad)

a.4) homogenized cheese/yogurt/kefir

a.5) egg

a.6) fast food (e.g. hamburger/hot dog)

a.7) others (e.g. sweets) ………………

b) Second breakfast:

b.1) sandwiches with ……………………

b.2) vegetables/fruit (e.g., in a salad)

b.3) homogenized cheese/yogurt/kefir

b.4) egg

b.5) fast food (e.g. hamburger/hot dog)

b.6) others (e.g. sweets) …………………

c) Dinner:

c.1) soup (what kind?..)

c.2) stuffed dumplings

c.3) meat dishes

c.4) fish

c.5) potatoes

c.6) rice

c.7) pasta

c.8) fruit/vegetable salad

c.9) fast food (e.g. gyros/pita/hamburger/hot dog)

c.10) others (e.g. sweets) …………………

d) afternoon snack:

d.1) sandwiches with ………………………………………

d.2) Vegetables/fruit (e.g. salad)

d.3) homogenized cheese/yogurt/kefir

d.4) fast food, e.g., hamburger/hot dog

d.5) others (e.g. sweets) …………………

e) supper:

e.1) sandwiches with ……………………………………….

e.2) Vegetables/fruit (e.g. salad)

e.3) pasta with …………………………………

e.4) rice with ……………………………………………

e.5) homogenized cheese/yogurt/kefir

e.6) fast food, e.g. hamburger/hot dog

e.7) others (e.g. sweets) …………………
